# Masculinity and Barebacker Identification in Men who have Sex with Men

**DOI:** 10.4172/2155-6113.1000276

**Published:** 2014-01-27

**Authors:** Christopher W. Wheldon, David L. Tilley, Hugh Klein

**Affiliations:** 1 Department of Community & Family Health, University of South Florida College of Public Health, Tampa, FL, USA; 2 Kensington Research Institute, Silver Spring, MD, USA

**Keywords:** MSM, Barebacking, Masculinity, Sexual behavior, Barebacker identity

## Abstract

**Purpose:**

Barebacking is a term that is used to refer to intentional involvement in unprotected anal sex. This paper examines the relationship between masculinity and self-identification as a barebacker, and how these factors related to HIV risk practices in a sample of men who have sex with other men (MSM).

**Method:**

As part of the Men4Men Study, a brief Internet-based survey was completed in 2007 with English-speaking MSM aged 18+ who were not involved in a marital/romantic relationship at the time of interview. 886 participants were recruited by placing electronic postings and banner advertisements on Weblogs, social and sexual networking sites, and listservs frequented by MSM.

**Results:**

A number of factors differentiated men who self-identified as barebackers from those who did not, and barebacking identity was linked with greater involvement in HIV risk practices. Multivariate analysis revealed that having a high level of masculinity was associated with a greater likelihood of self-identifying as a barebacker.

**Conclusions:**

HIV prevention and intervention efforts targeting MSM ought to address issues of self-identification as a barebacker as well as the extent to which men adhere to a masculine ideology.

## Introduction

Barebacking is a sexual behavior in which people, usually gay or bisexual men, intentionally engage in Unprotected Anal Intercourse (UAI). A growing body of research in public health and the social sciences is focused on improving our understanding of the barebacking phenomenon among men who have sex with men (MSM), which was first described in the gay press in the mid-1990s [1,2]. These empirical works have investigated emic understandings of barebacking [3–6], the role of the Internet in its proliferation [7,8], the prevalence of barebacking in a variety of gay and bisexual populations [5,9–14], as well as psychosocial (e.g., coping with social stressors) [13–15] and behavioral correlates (e.g., substance abuse) related to its practice [16].

Rather than resulting from episodic lapses in otherwise consistent condom use, barebacking is characterized by “intentional condomless anal sex in HIV-risk contexts” [17, p. 225]. The intentionality of UAI is emphasized in this definition, as it has been thought to represent a rejection of institutionalized safer-sex messages [18], and stands as a potential barrier to the prevention of HIV and other sexually transmitted infections among MSM. Furthermore, the reification of barebacking as a sociocultural practice is exemplified by recent studies that show subgroups of MSM, such as HIV-positive gay men, who identify as barebackers [11,19–21]. Although in-depth interviews with gay men in New York City did not yield evidence for barebacker as a dominant social identity [4], other analyses suggest that gay men may engage in barebacking as a means of constructing a sexual identity in opposition to heteronormative expectations of sexual behavior in the context of HIV/AIDS [18,22]. Dean argued that distinct bareback sexual subcultures exist within gay communities and that they are formed around expectations of sexuality that emphasize UAI as the object of identification [22]. In this way, “Bareback subculture reclaims gay sex as sexuality by relegating epidemiological concerns to secondary status” [22, p. 11]. The limited amount of research on barebacker identification indicates that approximately 12–31% of MSM self-identify as barebackers [11,19,21,23]. These estimates are far lower than the estimates of men reporting barebacking behavior [16], suggesting that self-identifying as a barebacker is something different than reporting barebacking behaviors and intentions. The reliability of these estimates is unknown, given that all of the studies sampled MSM in large urban centers with well-established gay communities. These studies do, however, provide preliminary data on sociodemographic, behavioral, and psychosocial correlates of barebacker identification.

Previous research has shown that, generally speaking, self-identified barebackers and other MSM do not differ on sociodemographic variables [11,19,21,24], aside from one study that found that the former were less likely to be college educated than non-barebackers [23]. Barebackers are also significantly more likely to be HIV-positive [19, 21,25]. In two community-based studies of urban gay and bisexual men, more than 1 HIV-positive man in 3 identified as a barebacker [19,21]. Problems with alcohol and recreational drug use appear to be more common among self-identified barebackers compared to other MSM [11,19,21,23,24]. Barebackers are also more likely to spend more time on the Internet looking for sex partners [19], and to engage in higher rates of intended and unintended UAI [19,21,23]. Barebackers also perceive greater psychological (e.g., “Barebacking is more ‘butch’ and manly”), emotional (e.g., “Barebacking increases intimacy between men”), and physical benefits (e.g., “Barebacking is ‘hotter’ than sex with condoms”) from UAI when compared to their nonbarebacking counterparts [25].

Berg [16] argued that to understand the motivations for barebacking, and subsequently design interventions to curtail or mitigate associated risks, more research is needed to examine the role of sociocultural factors. These factors, if left unaddressed, may act as barriers to lasting behavior change. Largely lacking in the existing empirical literature is a broader consideration of the sociocultural antecedents of barebacking as an emerging identity.

Some scholars have argued that barebacking is “gender-specific, and is tied to constructions and performances of masculinity, and to representations of risk” [26, p. 189]. In their analysis of popular Internet sites frequented by barebackers in New York City, Carballo-Diéguez and colleagues [8] found that interactions through these sites normalized barebacking behavior, while associating bareback sex with masculinity and courage. They observed that HIV risk taking discourses are presented “as something masculine men do,” whereas those who do not take such risks are associated with feminine characteristics [8, p. 485]. Masculinity, in this case, becomes a resource utilized by men to increase sexual pleasure and gratification within sexual contexts [27].

In addition to expressing normative expectations of male gender roles, the symbolic importance of masculinity is evident in descriptions of episodic sexual encounters by some barebackers [28]. Oftentimes, meanings ascribed to sex are organized around normative themes of masculinity. For instance, “barebacking could be a mode of ‘letting go’, be about muscles grinding, a means of celebrating masculinity and venturing beyond boundaries or feeling adventurous and free” [28, p. 275].

This body of research suggests that barebacking has, in part, emerged through a salient sexual subculture as a means of endorsing and constructing normative expectations of masculinity (i.e., masculinity ideology). Understood in this way, masculinity is defined culturally and embedded in social relations [29]. Connell’s concept of “protest masculinity” is helpful in understanding the process by which MSM construct masculinity in light of the common perception that male homosexuality is a negation of manhood [30]. By drawing upon normative expectations of masculinity, such as sexual prowess, stoicism, independence, and risk taking, scholars believe that marginalized men are able to structure their sexual interactions in order to gain a sense of status and increased sexual gratification [22,29,31]. Levine and Kimmel [31] argued that masculinity is constructed among MSM through the use of culturally-available sexual scripts that emphasize sexual conquest, emotional detachment, and the “pursuit of sexual gratification for its own sake, and by the association of danger and excitement” in sexual encounters (p. 156). These ideas are consistent with the observations made in the empirical studies previously mentioned. More research is needed, however, to investigate the association of barebacker identification and masculinity among larger and more diverse samples of MSM.

The purpose of the present study was to assess the association between normative masculinity and barebacker identification. We hypothesized that men who endorsed normative standards of masculinity would be more likely to self-identify as a barebacker after controlling for demographic and behavioral differences between identified barebackers and non-identified barebackers.

## Methods

### Participants and procedures

Data for the current analysis were collected during the spring and summer of 2007 as part of the Men4Men Study, a brief (10–15 minute) cross-sectional Internet-based survey, sampling English-speaking adult MSM aged 18 and older. Participants were recruited by placing electronic postings and banner advertisements on Weblogs, social and sexual networking sites, and listservs frequented by MSM. A wide variety of Internet-venues were selected in order to maximize the diversity of respondents with regard to demographic and behavioral characteristics. The administrators of these sites were contacted and asked to post an advertisement or description of the study as well as a link to the study’s website. Participants were directed to the study website by clicking on the link. Upon providing consent, participants immediately began the questionnaire. No compensation was offered. The University of South Florida’s institutional review board approved the objectives and methods of this study.

In order to be included in the study, men had to report having had sex with another man at least once during the previous 12 months. Additionally, because the predictors of UAI within the context of romantic relationships may differ from similar behaviors with casual partners, only men who were not in a primary relationship were eligible to participate. A total of 1,399 respondents met eligibility criteria. Of these, 513 (37%) exited the survey prior to completion. The dropout rate in this study is less than similar studies of Internet-based sexuality research [32,33]. The analysis reported here utilized data from 886 respondents with complete questionnaires. Compared to those who completed the survey, dropouts were younger (33 vs. 37 years old, *p* < 0.05), more likely to be bisexual (4% vs. 2%, *p* < 0.05), and more likely to have graduated college (39% vs. 30%, *p* < 0.05).

### Measures

#### Masculinity:

This research conceptualizes the sexual practices and subsequent identification with a sexual subculture as resources utilized by gay and bisexual men to construct normative or hegemonic masculinity. Masculinity, in this way, is understood as culturally defined and acted upon by individuals through performative aspects of behavior and identity. In order to quantitatively measure the patterns and effects of normative masculinity among larger groups of men, the concept of masculinity ideology was used in this study [34]. Masculinity ideology, which is used interchangeably with the term “normative masculinity” throughout the present paper, is defined as the “beliefs about the importance of men adhering to culturally defined standards for male behavior” and is operationalized by measures of “attitudes toward the male gender role” [34, p. 19]. Congruent with a social constructionist account of masculinity, instruments designed to quantify normative masculinity measure an individual’s endorsements of the dominant ideologies encompassing culturally “appropriate” behavior for men. In other words, normative masculinity is a measure of an individual’s endorsement of hegemonic norms.

A revised version of the Masculinity, Attitudes, Stress, and Conformity (MASC) questionnaire was used to assess normative masculinity in this study [35]. The MASC was developed in order to measure attitudes toward traditional male norms as well as an individual’s own conformity to and distress resulting from male role expectations. Only the subscale measuring attitudes towards masculinity was used in this study. The MASC attitudes scale consists of a total 36 items measuring six theoretical constructs (emotional restrictiveness, independence, achievement, dominance, aggressiveness, and sexuality), with each construct being measured by six items. Two of the original 36 items were not included in the current study because they were inappropriate for use with MSM. Therefore, the revised scale used in the current study included a total of 34 items; each measured using a six-point ordinal response scale ranging from “strongly disagree” to “strongly agree.” Higher scores indicated stronger endorsement of normative standards of masculinity. Scores on the composite measure of normative masculinity ranged from 1 to 6, with higher scores indicating stronger endorsement of normative masculinity. The MASC attitudes scale has demonstrated good internal consistency (α = 0.95) in previous research [35] as well as in the current sample (α = 0.94).

#### Sexual behavior:

Sexual behavior was measured by asking several questions about anal intercourse during the previous six months. Respondents were asked separately about receptive and insertive anal intercourse, including the frequency of HIV serodiscordant unprotected anal intercourse or unprotected anal intercourse with a partner(s) of unknown HIV status. For example, if a respondent indicated that he had receptive anal intercourse in the previous six months, he was asked to report with how many different men did this occur, with how many of these men did he engage in receptive anal intercourse without a condom, and also how many times he engaged in this behavior over the last six months. Two separate items asked if, in the last six months, he had receptive unprotected anal intercourse with a man whose HIV serostatus was different than his own or with a man for whom he was unaware of his HIV status. An identical set of items was asked separately regarding insertive anal intercourse. From this set of items, four binary variables were created to indicate any instance of (1) insertive unprotected anal intercourse, (2) receptive unprotected anal intercourse, (3) insertive unprotected anal intercourse with a partner of unknown or discordant HIV status, and (4) receptive unprotected anal intercourse with a partner of unknown or discordant HIV status. Respondents were also presented with a checklist of venues (i.e., “sex club or bathhouse,” “park or outdoor cruising location,” and “leather, fetish, BDSM bar or event”) and asked to indicate separately if they have ever had casual and/or anonymous sex with a man at that venue.

#### Barebacker identification:

Participants responded to a single barebacker identification item after they were provided with the following definition: “Barebacking has been described as intentionally having anal sex without a condom.”They were then provided with five statements (i.e., “I purposely seek out bareback sex as a top,” “I purposely seek out bareback sex as a bottom,” “I don’t seek out bareback sex, but if it happens that’s okay if I’m the top,” “I don’t seek out bareback sex, but if it happens that’s okay if I’m the bottom,” and “Bareback sex is only okay if I know for certain the other guy is the same HIV status as me”) regarding their intentions to bareback [19], followed by one item asking how strongly they agreed or disagreed with the following statement: “I consider myself a barebacker”[19]. A five-item response scale was provided that ranged from “strongly disagree” to “strongly agree” with a neutral midpoint (“neither agree nor disagree”). A binary measure of barebacker identification was created to contrast men who indicated, “Strongly agree” or “agree” to the single barebacker identity item (i.e., “I consider myself a barebacker”) from men who disagreed or were neutral. This measure of barebacker identification is similar to that used in previous research [19,21,23,24].

#### Internet use:

One item measured the frequency of using the Internet for meeting sexual partners within the previous six months. Responses were recorded on a 6-point scale including “not at all,” “1–2 times,” “3–9 times,” “10–19 times,” “20–49 times,” or “50 or more times.”A dichotomous measure was created to differentiate men who did (i.e., at least on 1 occasion) and did not (i.e., “not at all”) use the Internet to meet sex partners.

#### Substance use:

Participants were asked whether or not they had used any of the following substances immediately before or during sexual activity during the previous six months: “Crystal Meth (a.k.a. Tina, Crystal, Ice),” “Ecstasy,” “Ketamine (a.k.a. Special K),” “Poppers,” or “GHB.”For analytical purposes, this information was converted to a single dichotomous measure indicating whether or not men had used any of these substances in conjunction with sexual activity during the prior six months.

#### Personal characteristics:

Participants were asked to enter their age in years (continuous measure), race/ethnicity (coded as “White/Caucasian,” vs. nonwhite), sexual identity (gay, bisexual, heterosexual, or other), and the highest level of education completed (less than a high school diploma, high school diploma, some college, four-year college degree, or graduate school). Participants were also asked if they ever tested positive for HIV (yes/no).

### Analytic strategy

SAS version 9.2 was used for all analyses. Results were considered to be statistically significant whenever *p* < 0.05. First, bivariate analyses were conducted in order to examine the associations between each explanatory variable and barebacker identification. Only those variables that were associated significantly with barebacker identification were included in multivariate analyses. And while unprotected anal intercourse was associated significantly with barebacker identification (*p* < 0.05), only instances of serodiscordant unprotected anal intercourse were used as indicators of sexual risk behavior in the final models in order to account for the greatest risk of HIV transmission. This was done so as to avoid issues pertaining to multicollinearity and to be consistent with previous research [23].

All predictors were added to the models as dichotomous variables aside from age and the composite measure of normative masculinity, which were initially analyzed as continuous predictors. Modeling predictors as continuous variables assumes linearity of effect across the whole distribution of the predictor variable. For this reason, the linearity of the logit was examined using the Box and Tidwell method for which interaction terms for age and masculinity were created by multiplying each individual’s response with its natural logarithm [36]. The interaction term representing age was not significant (Wald *χ*^2^= 0.163, *p* = 0.686), but it was significant for masculinity (Wald *χ*^2^= 14.77, *p* < 0.001). Given this evidence for non-linearity in the logit, the composite index of masculinity was categorized into tertiles representing low endorsement, moderate endorsement, and high endorsement of normative standards of masculinity.

A series of sequential logistic regression models were estimated in order to assess the unique contribution of normative masculinity in predicting barebacker identification above and beyond the predictive power of sociodemographic and behavioral factors. The probability of barebacker identification was modeled with three nested logistic regression models. Personal characteristics (age, educational attainment, race/ethnicity, and HIV-serostatus) were entered as the first model, sexual risk behaviors (serodiscordant UAI, drug use, and history of sexual venue use) were added in the second model, and masculinity was added to the final model.The overall contribution to the prediction of barebacker identification between each successive model was evaluated using the likelihood ratio test. The likelihood ratio test uses the chi-square distribution with *k* degrees of freedom to test the statistical differences between the log likelihood functions of nested multivariable models. When this difference is statistically significant, the addition of the variable (or set of variables) under consideration is said to have statistically improved the overall fit of the model. The nested models include personal characteristics (Model 1), sexual behaviors (Model 2), and normative masculinity (Model 3), with the full model (i.e., Model 3) including all of the variables examined in Models 1 and 2.

## Results

### Sample characteristics

Characteristics of the study sample (*N* = 886) are reported in Table 1. Respondents were on average 36 years old (*SD* = 12). They were primarily white (83%) and college educated (60%). The majority of respondents identified as gay (98%). Approximately 8% reported testing positive for HIV and 5% reported having contracted a sexually transmitted infection within the previous six months (Table 1).

### Sexual risk behaviors

Involvement in sexual risk behaviors was not uncommon among participants. More than one-half of respondents reported ever having had sex with a partner first met on the Internet (63%), and approximately one-third ever had casual sex at a sex club/bathhouse (38%) or an outdoor cruising spot (32%). Ever having had casual sex at leather, fetish, BDSM bars or events was less common (11%). Insertive or receptive unprotected anal intercourse (IUAI and RUAI, respectively) within the previous six months was reported by 30% and 25% of respondents, respectively. Fewer men reported IUAI (15%) or RUAI (14%) with a partner of discordant or unknown HIV status. The number of respondents who identified as a barebacker was low (*n* = 88, 10%) even though 44% of the respondents indicated that they had engaged in unprotected insertive and/or receptive anal sex during the previous six months.

### Characteristics associated with identifying as a barebacker

There were significant bivariate differences between men who did and did not identify as a barebacker (Table 1). Self-identified barebackers were slightly older (*M* = 39, *SD* = 12; *t* = −2.41, *p* = 0.013) than nonbarebacker-identified men (*M* = 36, *SD* = 12). They were also more likely to report a high school education or less compared to nonbarebacker-identified men (58% vs. 38%; χ*2* (1*df*) = 12.49, *p* < 0.001). Barebackers, compared to nonbarebacker-identified men, were more likely to be Caucasian (92% vs. 82%; *χ2* (1*df*) = 5.82, *p* = 0.016) and to have tested positive for HIV (30% vs. 5%; *χ2* (1*df*) = 67.54, *p* <0.001). A larger proportion of barebackers reported a recent occurrence of IUAI (61% vs. 27%; χ2(1*df*) = 44.83, *p* <0.001), RUAI (61% vs. 21%; *χ2*(1*df*) = 69.86, *p* <0.001), serodiscordant IUAI (40% vs. 13%; χ2(1*df*) = 45.54, *p* <0.001), and serodiscordant RUAI (42% vs. 10%; *χ2* (1*df*) = 63.45, *p* <0.001). Furthermore, self-identified barebackers were more likely to report drug use during sexual activities (49% vs. 25%; *χ2*(1*df*) = 22.85, *p* <0.001), and to have had casual sex at a sex club/bathhouse (50% vs. 37%; *χ2*(1*df*) = 5.70, *p* =0.017), outdoor cruising spot (55% vs. 29%; *χ2*(1*df*) = 23.24, *p* <0.001), or leather/fetish bar (22% vs. 10%; *χ2*(1*df*) = 11.35, *p* <0.001).

On average, respondents moderately or slightly disagreed with the items measuring masculinity (*M* = 2.69, *SD* = 0.80). When divided into tertiles, the highest tertile had a moderate endorsement of normative masculinity (*M* = 3.56, *SD* = 0.50) compared to the middle (*M* = 2.66, *SD* = 0.20) and lowest tertile (*M* = 1.83, *SD* = 0.34). Chi-square test of independence indicated a significant association between masculinity and barebacker identification (χ*2*(2*df*) = 12.87, *p* = 0.002). Men who expressed high endorsement (50%) of normative masculinity were more likely to identify as a barebacker compared to men with low endorsement (24%) or moderate endorsement (26%). The latter two groups were combined for multivariable analysis given the similar relationship with barebacker identification.

Nested logistic regression models were used to determine if normative masculinity contributed to the prediction of barebacker identification beyond the explanatory power of personal and behavioral factors (Table 2). In Model 1, personal characteristics entered included age, education, race/ethnicity, and HIV-status and sufficiently discriminated between barebacker and nonbarebacker-identified men (χ*2* (4*df*) = 62.51, *p* < 0.001). Model 2 included the addition of sexual behaviors such as serodiscordant IUAI and RUAI, using drugs immediately before and/or during sexual activity, and ever having casual sex at a sex club/bathhouse, outdoor cruising spot, or fetish bar/event. A comparison of the log-likelihood ratios for Models 1 and 2 indicated that the prediction of barebacker identity was improved significantly with the addition of sexual behaviors (χ*2* (6*df*) = 45.81, *p* < 0.001). Similarly, the addition of normative masculinity resulted in statistically significant improvement in Model 3 over Model 2 (χ*2* (1*df*) = 11.02, *p* < 0.001), indicating that Model 3 represents the best model given the available data to predict barebacker identification.

In the final model, self-identified barebackers were found to be less likely to have graduated from college (*AOR* = 0.41, 95% *CI* = 0.24–0.67) and more likely to be HIV-positive (*AOR* = 2.95, 95% *CI* = 1.48–5.86) compared to their peers who did not consider themselves to be barebackers. They were also much more likely to have engaged in receptive (*AOR* = 2.69, 95% *CI* = 1.49–4.87) and insertive (*AOR* = 2.58, 95% *CI* = 1.47–4.54) UAI with a serodiscordant partner within the previous six months. Of the three sexual venues included in the model, self-identified barebackers were more likely than nonbarebackeridentified men to have used parks or outdoor cruising spots for casual sex (*AOR* = 2.26, 95% *CI* = 1.28–3.98). Finally, normative masculinity significantly differentiated between barebackers from nonbarebackers. Men who endorsed higher standards of normative masculinity had more than twice the odds of self-identifying as barebackers compared to men who endorsed lower or moderate levels of normative masculinity (*AOR* = 2.41, 95% *CI* = 1.46–3.97).

## Discussion

Approximately 10% of men in this sample self-identified as barebackers, and these men were more likely than their nonbarebackeridentified counterparts to engage in a variety of high-risk sexual behaviors. Previous research estimates that anywhere from 12% to 31% of MSM self-identify as barebackers [11,19,21,23]. These estimates are slightly higher than that reported in the current study, possibly reflecting differences in sampling (largely, previous research was based on MSM in large urban cities) and/or measurement error.

As in previous research, barebackers in this sample were more likely to report lower education, to identify as non-Hispanic white, and to report serodiscordant UAI [11,23]. The degree to which self-identified barebackers are more likely to use drugs (such as poppers or club drugs like ecstasy) during sex has been somewhat equivocal in the extant literature. Some studies have found support for an overall higher risk profile among barebackers, including the use of illicit drugs and higher levels of alcohol and other drug-related sexual expectancies; however, these factors appear to be more salient among HIV-positive barebackers [11,21]. In fact, in the present study and in one previous investigation of HIV-negative MSM, the prevalence of drug use during sexual activity among barebackers and nonbarebackers was similar after controlling for other risk factors [23]. In the present research, we found that barebackers were more likely to have met partners at outdoor cruising locations, possibly suggesting a higher degree of sexual compulsivity among these men--an interpretation that has garnered support in previous research [11,21]. In this study, meeting sex partners from other sexual venues, such as the Internet, did not differ significantly among men who self-identified as barebackers and those who did not, contrasting with previously reported findings [19].

We also found that normative masculinity was associated positively with barebacker identity, even after controlling for demographic and behavioral correlates. This finding suggests that, for a subgroup of MSM, barebacking is more than a mere sexual practice and, indeed, appears to represent a cultural phenomenon that is actively engaging gender ideologies. Bareback sex occurs in a variety of sociocultural and sociosexual contexts and it is the unique way that these various contexts interplay with one another that oftentimes determines the nature of the sexual acts practiced and the inherent levels of HIV risk involved. For example, whether men consider themselves to be “tops” or “bottoms” sexually--that is, the insertive or the receptive partner--frequently plays an important role in determining whether or not bareback sex occurs. Researchers have shown that, oftentimes, men who self-identify as “tops” consider themselves to be at lower risk for contracting HIV than do men who self-identify as sexual “bottoms” and, therefore, they are more likely to engage in barebacking behaviors [5,26]. Furthermore, oftentimes, these roles are understood as gendered expressions of masculinity and femininity that influence sexual practices among gay and bisexual men [37].

In the current study, however, we found that endorsement of normative masculinity had no association with either insertive or receptive anal intercourse. In other words, men who endorsed greater norms of masculinity were no more and no less likely to report being a “top” or a “bottom.” In their in-depth analyses of websites focusing on bareback sex, Dowsett and colleagues provided some contextual explanation for this divergent finding [27]. Users of the barebacking sites included in their study described the role of the receptive partner (i.e., the “bottom”) in very active and masculine terms. The receptive role, including the act of receiving semen from another man, was described as “manly” and “what real men do” [27, p. 127]. As such, men participating in barebacking as a sexual subculture appear to be engaging actively in the eroticism and masculinization of homosexual acts that are commonly construed as being feminine or emasculating.

In this context, the concept of protest masculinity may prove to be helpful in understanding barebacker identification--that is, men who identify with and consider themselves to belong to a sexual subculture that is centered around bareback sex--within the larger sociocultural context. In this case, protest masculinity refers to the exaggeration of hegemonic norms pertaining to gender-stereotyped behaviors, such as sexual prowess and risk taking, in response to social proscriptions against homosexual behavior [29,37]. If we conceptualize barebacking as a behavior used by a subset of MSM to define masculinity for themselves and to perform acts that they consider to be masculine, then social identification of oneself as a barebacker may lead to the formation of a subculture within established gay communities. The act of identifying and involving oneself with this subculture, even if only through online websites, could, thus, be understood as a rejection of cultural representations of homosexual desires and behaviors as emasculating (as well as a rejection of institutionalized safer sex messages). If this interpretation is correct, then we would expect to find that men who identify with this subculture would adopt similar ideological views of masculinity. In this regard, the present study has provided empirical support for this hypothesis. Nevertheless, the degree to which barebacking as a social identity that has emerged within large urban areas with established gay subcultures requires further investigation.

Given the substantial disparities in HIV infection rates among various subpopulations of MSM, our lower-end estimate of barebacker identification and the associated high-risk behavioral patterns indicate that there is a sizable population that could benefit from targeted HIV prevention. The greater odds of HIV infection and serodiscordant UAI among men who self-identified as barebackers suggest that this group of men represents a high-risk subpopulation of MSM. The greater endorsement of cultural standards of masculinity among this group also suggests that their identification as barebackers and their greater involvement in sexual risk behaviors may be embedded in sociocultural processes that are resistant to--perhaps even in opposition to--safer sex messages promoting behavior change. Alternative prevention approaches, such as the use of pre-exposure prophylaxis (PrEP), may be of particular benefit for this subgroup of HIV-negative MSM. For HIV-positive barebackers, the promotion of harm reduction strategies, such as serosorting and strategic positioning, may be more tenable than promoting condom use.

The results of this study should be interpreted with caution given the limitations of the study methodology. This non-probability sample was highly educated, mostly gay identified, and white/Caucasian, thereby preventing generalizations to the broader population of MSM. The majority of the sample was recruited from weblogs and online networking sites and therefore may be more representative of established Internet users than of MSM in general. Without knowing participation rates, generalizability cannot be established. Likewise, it is impossible to know why such a large proportion of the people who began the survey failed to complete it. There were differences between the men who completed the survey and those who did not, suggesting at least some bias in the representativeness of the study population.

Despite these limitations, this study makes a unique contribution to the behavioral literature regarding HIV prevention in MSM and offers new insights for primary prevention. The exploration of sociocultural factors, such as the impact of dominant gender ideologies and emerging social identities, can expand upon our understanding of HIV-related risk behaviors. Future research should explore these factors using mixed methods research designs, and they should consider adopting more in-depth analyses. Additional qualitative research with diverse populations of MSM would be particularly informative, given the complexity and widespread variation in the development and expression of social identities. In addition, this type of research could offer more insight into how identifying as a barebacker might structure sexual interactions in ways that increase the risk of HIV transmission.

## Figures and Tables

**Table 1:** Personal characteristics, sexual risk behaviors, and normative masculinity by barebacker identification (N = 886).

	Total Sample		Barebacker Identification				
			Yes (n = 88)		No (n = 798)		
	*n*	(%)	*n*	(%)	*n*	(%)	*p*-value
**Mean age (*SD*)**	35	(11)	39	(12)	36	(12)	0.013
							
**Education**							<0.001
High school graduate or less	358	(40)	51	(58)	307	(38)	
College graduate	528	(60)	37	(42)	491	(62)	
							
**Race/Ethnicity**							0.016
White	734	(83)	81	(92)	653	(82)	
Non-white	152	(17)	7	(8)	145	(18)	
							
**Sexual identity**							0.641
Gay	872	(98)	86	(98)	786	(98)	
Bisexual/other	14	(2)	2	(2)	12	(2)	
							
**Sexual health**							
HIV positive	67	(8)	26	(30)	41	(5)	<0.001
STI (past 6 months)	40	(5)	6	(7)	34	(4)	0.275
							
**Sexual risk behavior (past 6 months)**							
Insertive UAI	268	(30)	54	(61)	214	(27)	<0.001
Receptive UAI	220	(25)	54	(61)	166	(21)	<0.001
Serodiscordant insertive UAI	135	(15)	35	(40)	100	(13)	<0.001
Serodiscordant receptive UAI	119	(13)	36	(41)	83	(10)	<0.001
Drug use during sexual activities	242	(27)	43	(49)	199	(25)	<0.001
Had sex with partner first met online	557	(63)	58	(66)	499	(63)	0.534
							
**Ever had casual sex at these venues**							
Sex club/bathhouse	339	(38)	44	(50)	295	(37)	0.017
Park/Outdoor cruising location	282	(32)	48	(55)	234	(29)	<0.001
Leather, fetish, BDSM bar or event	97	(11)	19	(22)	78	(10)	<0.001
							
**Normative masculinity**							0.002
Low endorsement	291	(33)	21	(24)	270	(34)	
Moderate endorsement	303	(34)	23	(26)	280	(35)	
High endorsement	292	(33)	44	(50)	248	(31)	

Note. UAI = Unprotected anal intercourse

**Table 2:** Nested logistic regression models of barebacker identification on personal characteristics, sexual risk behaviors, and normative masculinity (N = 886).

	Model 1	Model 2	Model 3
**Personal characteristics**			
Age	1.02 (0.99,1.04)	1.01 (0.99,1.04)	1.01 (0.99,1.04)
College graduate (yes vs. no)	0.44 (0.28,0.71)	0.40 (0.25,0.68)	0.41 (0.24,0.67)
Non-Hispanic White	2.25 (0.99,5.12)	2.27 (0.98,5.27)	2.30 (0.99,5.35)
HIV-positive	6.53 (3.66,11.63)	2.88 (1.46,5.69)	2.95 (1.48,5.86)
**Sexual risk behavior (past 6 months)**			
Serodiscordant insertive UAI		2.59 (1.48,4.53)	2.58 (1.47,4.54)
Serodiscordant receptive UAI		2.85 (1.59,5.10)	2.69 (1.49,4.87)
Drug use during sexual activities		1.37 (0.79,2.37)	1.31 (0.76,2.28)
**Ever had casual sex at these venues**			
Sex club/bathhouse		0.74 (0.41,1.35)	0.71 (0.39,1.30)
Park/Outdoor cruising location		2.12 (1.21,3.72)	2.26 (1.28,3.98)
Leather, fetish, BDSM bar or event		1.03 (0.50,2.15)	1.06 (0.51,2.22)
**Normative masculinity (high vs. low/moderate)**			2.41 (1.46,3.97)
			
**Model Chi-square (*df*)**	62.51 (4)	108.33 (10)	119.35 (11)
-2 log L	510.89	465.08	454.06
Δ -2 log L		45.81^a^	11.02^b^

*Note*. Values are adjusted odds ratios (95% CI); bolded values indicate statistically significant Wald Chi-square (*p*< 0.05)

a G = 510.89 – 465.08 = 45.81, distributed as Chi-square with 10 – 4 degrees of freedom, *p*<0.001

b G = 465.08 – 454.06 = 11.02, distributed as Chi-square with 11 – 10 degrees of freedom, *p*<0.001
